# Safety, quality attributes, and health risk assessments of sand smelt fish

**DOI:** 10.1038/s41598-025-03323-x

**Published:** 2025-05-25

**Authors:** Gehad A. Ezzat, Gehan M.A. Kassem, Nermeen M.L. Malak

**Affiliations:** Department of Food Hygiene and Control, Faculty of Veterinary Medicine, Cairo University, Giza, 12211 Egypt

**Keywords:** Sand smelt, *Atherina boyeri*, Health risk assessment, Fat and protein oxidation, Bacterial safety, Heavy metal content, Microbiology, Zoology, Natural hazards, Ocean sciences, Diseases, Health care, Health occupations, Risk factors

## Abstract

This study investigated the nutritional values, microbiological quality, heavy metal content, and their health risk assessment in sand-smelt fish (*Atherina boyeri*) in Egyptian markets. Fifty samples of sand-smelt fish were obtained from fish markets in Cairo and Giza Governorates. Fish samples were exposed to chemical analysis (protein, fat, moisture, and ash content), deterioration criteria [pH, Total volatile basic nitrogen (TVB-N), Trimethylamine (TMA), Thiobarbituric acid (TBA), Free Fatty Acids (FFA), Acid Number content (AN)], microbial testing (aerobic bacterial count (APC), psychrotrophic, *Enterobacteriaceae*, total coliforms, faecal coliforms, pseudomonas, and Aeromonas species), determination of heavy metals and their health risk assessment parameters. The study revealed that sand-smelt fish meat is rich in protein (18.53%), and contains a low-fat content (1.70%). Also, it contains reasonable levels of moisture (78.10%) and ash (1.61%). Furthermore, the study found low levels of the total bacterial count, psychrotrophic, *Enterobacteriaceae*, total coliforms, faecal coliforms, pseudomonas, and Aeromonas species (5.33, 4.56, 3.88, 4.50, 3.75, 2.73, 2.63 log_10_ CFU/g, respectively), indicating good microbiological quality. Moreover, fish muscles had good shelf life indicators and they met the Egyptian standard specifications in terms of pH, TBVN, TMA, TBA, FFA, and AN (6.27, 15.96 mg/100 g, 8.61 mg/100 g, 0.34 mg MDA/kg, 0.68% as Oleic acid, 1.31 mg NaOH/g, respectively). Regarding heavy metal content (µg/g) in fish meat, lead (1.27), arsenic (0.70), and cadmium (0.27) were detected, their levels were generally low. Mercury levels were below the detectable limits. Target Hazard Quotient (THQ) and Hazard Index (HI) values were below 1, suggesting a low risk of non-carcinogenic effects. Carcinogenic risks were also considered low. Interestingly, sand smelt fish meat can be widely incorporated safely in Egyptian markets due to its high nutritional value, safety, and quality indicators, besides its affordable price. However, continuous monitoring of heavy metal levels is recommended to ensure long-term food safety.

## Introduction

Sand smelt fish (*Atherina boyeri*) are an important part of aquatic ecosystems and a valuable food source for many cultures. It is small, short-lived fish, and is widely distributed in the Mediterranean, adjacent seas, and the northeastern Atlantic^1,2^. Sand smelt fisheries are a significant commercial activity along the Croatian coast, particularly in the western regions of the Istrian peninsula and its estuaries. It thrives in diverse aquatic environments, from coastal and estuarine waters to lagoons, salt marshes, and even inland waters^3^. Despite its tolerance for a wide salinity range, it is primarily carnivorous, feeding on zooplankton and small bottom-dwelling organisms. Reproduction occurs in brackish or hypersaline waters during spring and early summer months. Sand smelt has been accidentally introduced to various inland water bodies in Turkey, such as rivers, lakes, and reservoirs. This invasive species has thrived in these new environments due to its high adaptability and rapid reproduction rate^4^. Initially reported in Sapanca Lake, sand smelt has spread widely across Turkey. It is currently one of the region’s most commercially valuable invasive fish^5^.

Sand smelt is abundant in unsaturated fatty acids, protein, and essential vitamins and minerals, making it an excellent addition to a balanced diet^6^. In Egypt, sand smelt, locally referred to as “bissaria,” holds a significant presence due to its affordability and is widely distributed in fish markets, yet its profitability remains limited^7^. It is commonly used for the preparation of a traditional and seasonal dish in Egypt known as “moloha.” This type of salted fish is favored by numerous individuals in Upper Egypt, especially those facing financial difficulties^6^. Saedy moloha is typically made using small freshwater fish such as sand smelt fish (some sand smelt migrate to rivers from the sea for breeding, while others live entirely in freshwater)^8^. Additionally, this fish was previously used for processing some fish products such as fish fingers^2^.

Assessing the microbiological quality of fresh and frozen fish is crucial for ensuring food safety. Premium fresh fish typically exhibit microbial counts ranging from 3 to 4 log CFU/g, with higher counts observed in gills and intestinal tracts^9^. However, contamination of aquatic environments, especially in Egypt, poses significant environmental challenges due to heavy metal accumulation from various sources, including agricultural, household, and industrial effluents^10^. The bioaccumulation of metals in aquatic organisms, including fish, poses health risks to consumers, with metals like lead, mercury, cadmium, and arsenic being particularly toxic, even at low concentrations^11–13^. While certain heavy metals like iron, copper, cobalt, nickel, manganese, and zinc are essential for biological functions, excessive intake can lead to toxicity^14^. Therefore, understanding the dynamics of heavy metal accumulation in fish and their potential health implications is crucial for both environmental conservation and public health.

However, there is a lack of comprehensive information on their nutritional composition, microbiological safety, and potential heavy metal contamination. This study aims to address this knowledge gap by investigating the nutritional value, microbiological quality, and heavy metal levels of sand-smelt fish. By providing scientific evidence, we hope to inform consumers, policy makers, and the fishing industry about the benefits and potential risks associated with consuming sand-smelt fish. Our findings will contribute to the development of sustainable fishing practices and effective food safety regulations to provide insights into its suitability for consumption and address potential health concerns associated with metal exposure.

## Material and procedures

### Collection of samples

**Fig. 1:**
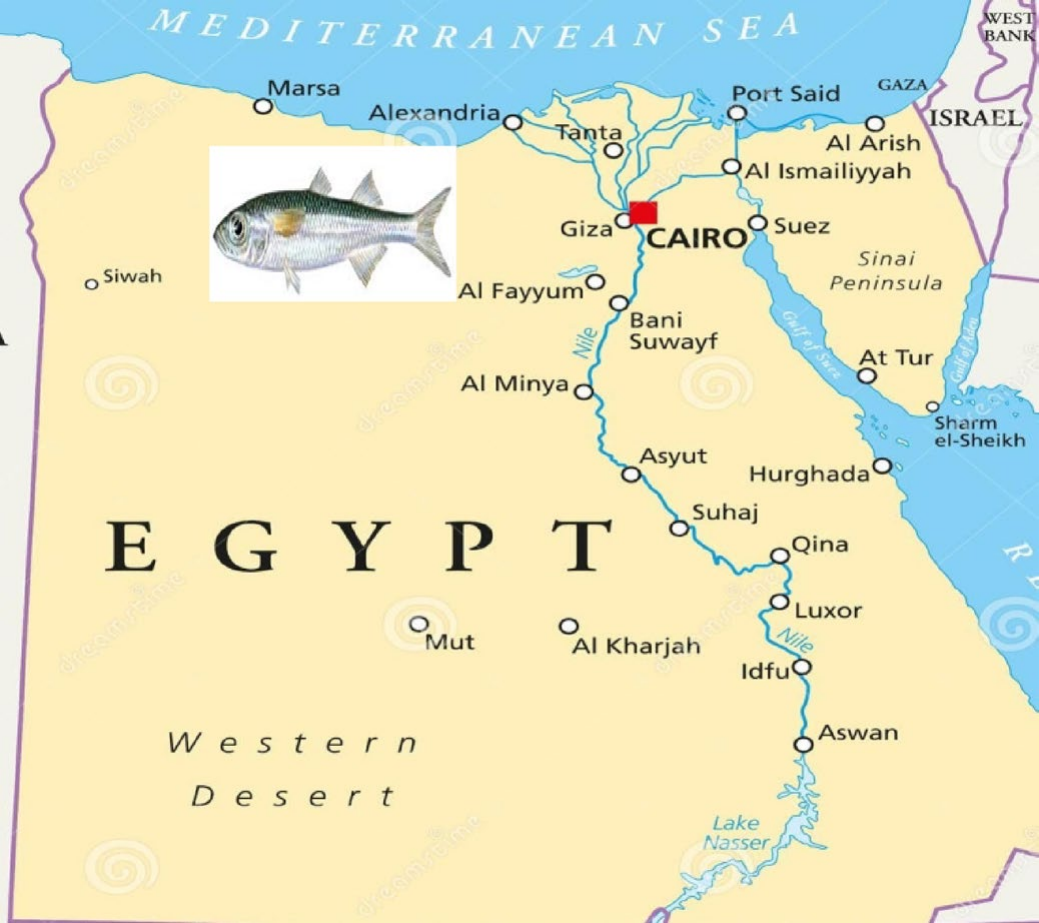
Study map of sand smelt fish (*Atherina boyeri*) collection areas from fish markets in Cairo and Giza governorates.

Fifty samples of sand smelt fish (about 500 g) were obtained from several fish markets in Cairo and Giza Governorates, Egypt (Fig. 1). Samples were immediately transferred into insulated ice boxes. After washing and removing blood clots or bones, samples were put into impermeable bags, labeled, and stored at 0–2 ºC until evaluation.

## Examinations

### Microbiological examination

Ten grams of fresh sand smelt fish meat were aseptically taken and homogenized with 90 mL of sterile Ringer’s solution (Oxoid BR0052G, Hampshire, England) in a sterile homogenizing bag using a stomacher (Seward 80 Lab Blender, compact, 110 VAC) for 2 min to achieve a dilution of 10^−1^. From this, ten fold serial dilutions were prepared. The aerobic plate count (APC) was enumerated by spreading 0.1 mL from the diluted tubes onto the surface of double sets of Plate Count Agar (PCA, Oxoid CM0325B, Hampshire, England), then kept in incubator (Heraeus B5050E Incubator) at 35 °C for 48 h^16^. Another set of inoculated plate count agar was incubated at 7 °C for 7–10 days to enumerate psychrotrophic bacteria^16^. *Enterobacteriaceae* bacterial counts were enumerated by incubating the inoculated Violet Red Bile Glucose agar plates (VRBG, Oxoid CM1082B, Hampshire, England) at 37 °C for 24 h^17^.

Additionally, for the enumeration of Pseudomonas and Aeromonas bacteria, double sets of Glutamate Starch Phenol-red agar plates were inoculated and incubated for 3 days at 25 °C^18^. Coliforms were counted following the method described by APHA^19^, where 1 mL of each prepared dilution was inoculated in lauryl sulphate tryptose broth and incubated for 24–48 h at 37 °C. Subsequently, 1 mL of positive tubes was transferred to a lauryl sulphate tryptose tube and incubated for 48 h at 44.5 °C. Coliforms and faecal coliforms were counted using the most probable number table^20^. For the enumeration of *E. coli*, a loop from positive tubes was streaked on Eosin Methylene Blue media (EMB) after incubation for 24 h at 37 °C.

## Physicochemical analyses

### Measurement of pH

The pH value was determined following the procedure outlined by Lee and Shin^21^ using a digital pH meter (Lovibond, Senso Direct). Initially, 5 g of fish meat samples were homogenized with 20 mL of distilled water. Subsequently, the pH meter was calibrated using 7.0 and 4.0 buffer solutions.

### Total volatile base nitrogen (TVB-N)

Total volatile base nitrogen (TVB-N) was assessed using the steam-distillation method as described by Malle and Tao^22^. Initially, fish extracts were prepared by homogenizing 100 g of the sample with 200 mL of a 7.5% trichloroacetic acid (TCA) aqueous solution (v/v) in a laboratory homogenizer at high speed for 1 min. The resulting homogenate was then centrifuged for 5 min at 3000 rpm, and the supernatant liquid was filtered through Whatman No. 1 filter paper. In a Kjeldahl-type distillation tube, 25 mL of the filtrate and 5 mL of a 10% NaOH (w/v) aqueous solution were combined. Steam-distillation was conducted, and 40 mL of the distillate were collected into a receiving beaker containing 10 mL of a 4% boric acid (v/v) aqueous solution and 0.04 mL of methyl red and bromocresol green indicator. The boric acid solution changed from pink to green upon alkalinization by the distilled TVB-N. Subsequently, the collected distillate was titrated with a sulfuric acid solution (0.1 N) until complete neutralization, indicated by a color change to pink. TVB-N values were then calculated from the volume of sulfuric acid (0.1 N) used for titration, multiplied by 16.8, and expressed in mg/100 g of sample.

### Trimethylamine acid (TMA)

The measurement of Trimethylamine (TMA) followed a similar procedure to that of TVB-N, with the only variation being the addition of 20 mL of a 16% formaldehyde (v/v) solution into the distillation tube. This addition aimed to block both primary and secondary amines, allowing only the tertiary amines to react. The results were expressed in mg/100 g of sample.

## Fat oxidation parameters

### Thiobarbituric acid (TBA)

TBA values were determined following the method proposed by Tarladgis et al.^23^. Initially, 10 g of fish samples were blended with 2.5 mL of 4 N HCl solution and 97.5 mL of distilled water for 2 min. The resulting blend was then loaded into a distillation tube, and steam-distillation was carried out until a volume of 50 mL of distillate was obtained. Five milliliters of the distillate were transferred into a test tube containing 5 mL of 0.02 M TBA reagent in acetic acid (90%), followed by heating for 35 min in a boiling water bath to produce the TBA-MDA chromogen. Afterward, the test tubes were cooled for 10 min under running tap water, and the absorbance was measured using a spectrophotometer (Unico 1200, USA) at 538 nm against a blank containing 5 mL of distilled water and 5 mL of TBA reagent. The amount of malonaldehyde (MDA) was calculated based on a standard calibration curve using various dilutions of 1,1,3,3-tetraethoxypropane. A conversion factor of 7.8 was applied to convert the TBA-MDA absorbance readings to TBA values, expressed as mg MDA/kg.

### Measurement of Free Fatty Acids content and Acid Number

The determination of free fatty acids (FFAs) and Acid Number (AN) in the samples followed the standard procedure outlined by AOCS official method^24^. Fifteen grams of sample were homogenized in 60 mL of chloroform and 60 mL of methanol solvents. After 24 h, 48 mL of water were added, and the oil was collected. The oil, extracted in the presence of phenolphthalein (1%), was titrated with sodium hydroxide (0.1 N).

### Proximate chemical composition

Fish meat samples were chemically examined according to the methodology established by the AOAC^25^. The samples were dehydrated in an oven (Heraeus Functionline T6) for 24 h at 105 °C, and then samples were weighed obtaining three successive constant weights. To get the moisture content in each sample, the difference between the weight of samples before and after drying in the oven was calculated. Moreover, one gram of dried samples was incinerated for 6 h at 550 °C in a muffle furnace (Thermo Scientific™ FD1530M) to evaluate their ash content. The crude lipid content was measured using a gravimetric technique by Soxhlet lipid extraction. Three grams of previously dried samples was put in a thimble and extracted by petroleum ether in a soxhlet extraction unit for six hours. Crude protein was quantified using the Kjeldahl method and a distillation apparatus. In digestion tubes, a half-gram from previously prepared samples mixed with potassium sulfate, 98% sulphuric acid, and copper sulfate was heated on a VELP Scintifica digester (DK 6) at 445 °C for 45 min. The digested samples were distilled in a receiving flask containing methyl red indicator and 4% boric acid, then titrated with hydrochloric acid solution to a red end-point. Protein content is estimated by multiplying nitrogen content by 6.25. The percentages of ash, fat, protein, and moisture were given as percentages.

### Measurement of heavy metal content

According to the EC, European Committee for Standardization^26^, a closed-system microwave was used to measure the concentration of heavy metals in the samples. After weighing, one gram of homogenized fish sample was placed in PTFE containers for microwave digestion. The material was then combined with 8 mL of 69% nitric acid and 2 mL of hydrogen peroxide. After carefully and completely closing the vessel, it was placed in the microwave to finish the digestion process. The digestion process was conducted utilizing a temperature-controlled microwave oven program, involving 15 min of holding, followed by heating to 200 °C for 15 min, and subsequent cooling to 85 °C for another 15 min. After the vessel cooled to room temperature, its contents were transferred into a 25-mL volumetric flask and diluted with ultrapure water for atomic absorption analysis. The analysis of the heavy metals of interest was performed using an atomic absorption spectrophotometer (ICE 3500 series) (thermal) at the Food Toxicology and Contaminants Department of the National Research Centre, following the methodology described by Abdel-Rahman et al.^27^.

### Health risk assessment parameters of heavy metals associated with consuming sand-smelt fish muscles

#### Estimated daily intake (EDI)

Estimated daily intake helps us to understand the potential long-term exposure to heavy metals from consuming this fish. The estimated daily intake (EDI) of heavy metals in sand smelt fish muscles was calculated based on a formula (1) suggested by the US Environmental Protection Agency (USEPA)^28,29^.

1 $$EDI{\text{ }}(mg/k{g^{ - d}}){\text{ }} = {\text{ }}\left( {Cm{\text{ }} \times {\text{ }}IR} \right)/Bwt$$

Where Cm is concentration of heavy metal in fish muscle sample (mg/kg); IR is the rate of ingestion (0.0555 kg/day for adults^30^. Bwt is the body weight (70 kg for adults according to USEPA^28^.

#### Target hazard quotient (THQ)

There are Two methods are used to assess health risks associated with heavy metal exposure: carcinogenic and non-carcinogenic. Non-cancer health risks, stemming from consuming fish contaminated with heavy metals, are evaluated using two indicators: Hazard Index (HI) and Target Hazard Quotient (THQ). THQ is calculated using a formula (2) provided by the US Environmental Protection Agency^31,32^. This formula compares the estimated daily intake (EDI) of a heavy metal to its oral reference dose (RfD). RfD values, which represent the daily exposure level likely to pose no adverse health effects, have been established for lead (Pb), cadmium (Cd), mercury (Hg), and inorganic arsenic (Ar) as follow: 0.004, 0.001, 0.0001, and 0.0003 mg/kg-d, respectively^28–33^.

2 $$THQ= EDI/RfD$$

#### Hazard index (HI)

Total potential health risk, or Hazard Index (HI), was calculated by adding up the individual Target Hazard Quotients (THQ) for each heavy metal detected in the fish samples. This calculation method is outlined in a formula presented by Rauf et al.^32^ and the US Environmental Protection Agency^34^.

3 $$HI = {\text{ }}\Sigma THQi$$

where i represents each metal.

#### Carcinogenic risk (CR)

The carcinogenic risk (CR) was calculated to estimate the likelihood of developing cancer over a lifetime due to exposure to heavy metals in fish. This calculation was based on a formula (4)^32^.

4 $$CR = EDI X CSFo$$

Where the CSFo (oral carcinogenic slope factor) and their values as follows: lead (Pb), cadmium (Cd), mercury (Hg), and inorganic arsenic (Ar) are 0.0085, 0.38, and 1.50 (mg/kg-d)^−1^, respectively^33,35^.

#### Statistical analysis

Each measurement was repeated three times, and SPSS Statistics 27.0 for Windows was used to evaluate the entire dataset. The data’s mean and standard deviation were determined.

## Results

### Nutritional value of sand smelt fish

**Fig. 2:**
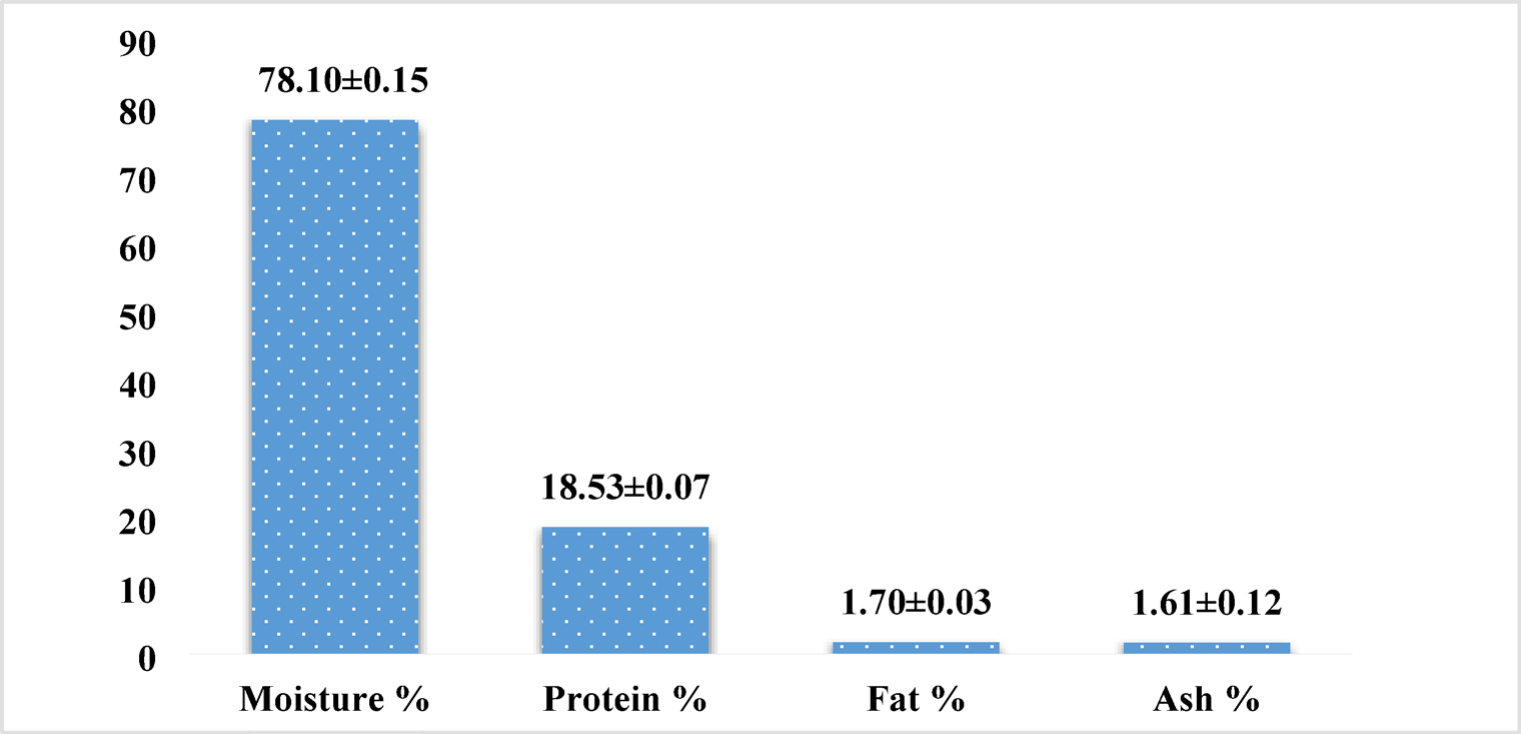
Chemical composition of raw sand smelt fish muscles (Mean values ± standard deviation).

Figure (2) shows the results of the proximate chemical analysis of raw sand smelt fish. The fish flesh contained approximately 78.10 ± 0.15% moisture, 18.53 ± 0.07% protein, 1.70 ± 0.03% fat, and 1.61 ± 0.12% ash.

### Bacterial load in the muscles of the examined fish samples

Table (1) summarizes the results of the bacteriological examination of fresh sand smelt fish muscles. The mean bacterial counts (log_10_ CFU/g) were as follows: aerobic bacterial count (APC) (5.33 ± 0.45), Psychrotrophes (4.56 ± 0.44), *Enterobacteriaceae* (3.88 ± 0.90), Total coliforms (4.50 ± 0.47), Faecal coliforms (3.75 ± 0.41), Pseudomonas spp. (2.73 ± 0.23), and Aeromonas spp. (2.63 ± 0.30).

**Table 1:** Bacteriological examination (log_10_ CFU/g) of examined Raw sand smelt fish.

Microorganisms	Mean ± SD	Minimum	Maximum
Aerobic bacterial count	5.33 ± 0.45	4.89	5.99
Psychrotrophes	4.56 ± 0.44	4.00	5.09
Enterobacteriaceae	3.88 ± 0.90	2.30	4.43
Total coliform	4.50 ± 0.47	4.00	5.00
Faecal coliforms	3.75 ± 0.41	3.00	4.00
Pseudomonas spp.	2.73 ± 0.23	2.90	3.00
Aeromonas spp.	2.63 ± 0.30	3.00	2.75

Values represent the mean of 3 independent replicates ± Standard Deviation.

### Protein deterioration and fat oxidation parameters of raw sand smelt fish muscles

The physicochemical quality attributes and fat oxidation parameters of sand smelt flesh are presented in Table 2. The mean values ± standard deviation were as follows: pH (6.27 ± 0.07), Total volatile basic Nitrogen (TVB-N) (15.96 ± 0.55 mg/100 g), trimethylamine (TMA) (8.61 ± 0.82 mg/100 g), thiobarbituric acid (TBA) (0.34 ± 0.01 mg MDA/kg), free fatty acids (FFAs) (0.68 ± 0.01% as oleic acid), and Acid Number (AN) (1.31 ± 0.23 mg NaOH/g).

**Table 2:** Protein deterioration and fat oxidation parameters of Raw sand smelt fish.

Parameters	Mean ± SD	Minimum	Maximum
pH	6.27 ± 0.07	6.18	6.35
TVB-N	15.96 ± 0.55	15.29	16.63
TMA	8.61 ± 0.82	7.60	9.62
TBA	0.34 ± 0.01	0.33	0.35
FFAs	0.68 ± 0.01	0.67	0.70
AN	1.31 ± 0.23	1.02	1.59

TVB-N: total volatile base nitrogen (mg/100 g); TMA: trimethylamine (mg/100 g); TBA: thiobarbituric acid (mg MDA/kg); FFA: free fatty acid % as Oleic acid; AN: Acid number (mg NaOH/g).

### Heavy metal levels in the examined sand smelt fish muscles

**Fig. 3:**
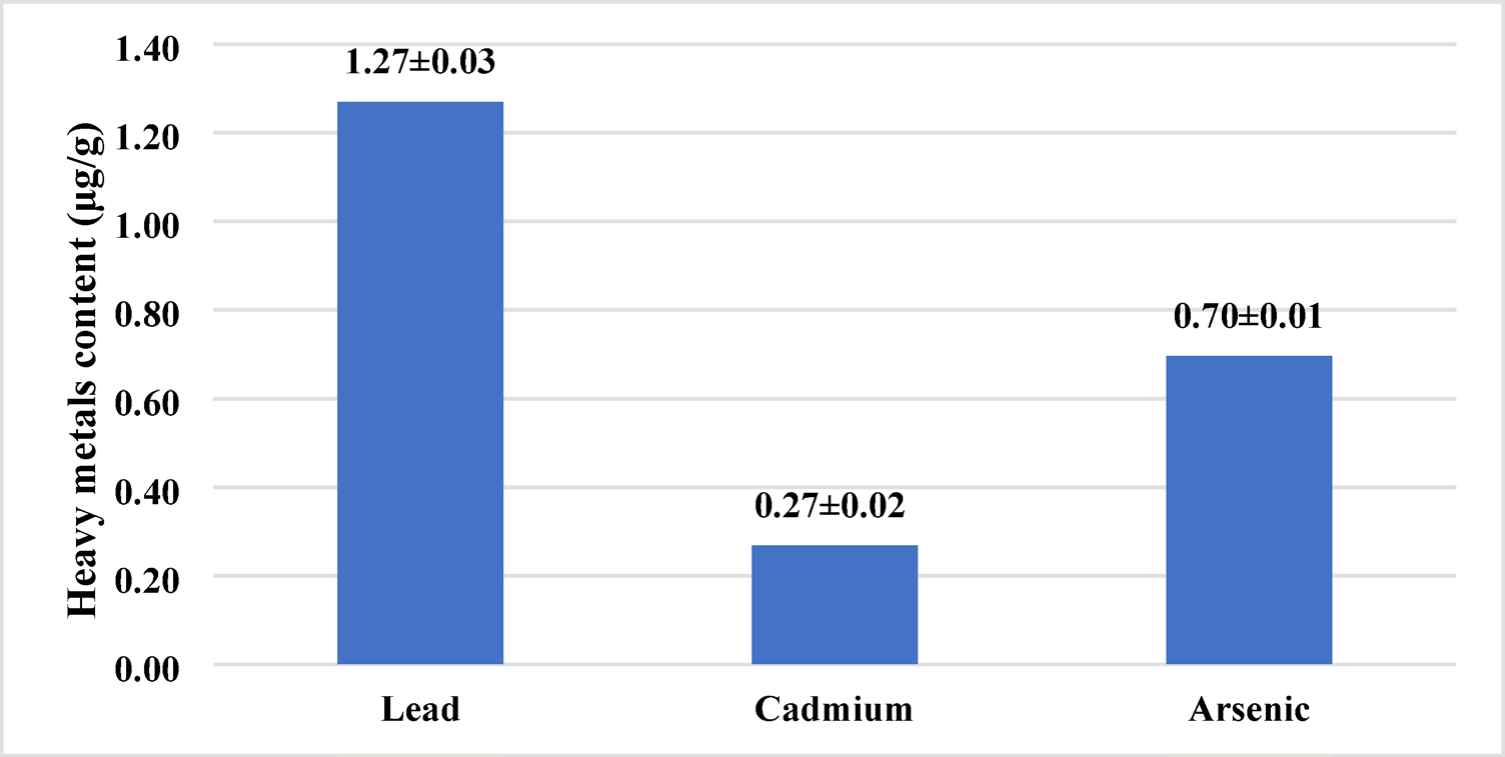
Heavy metals content (µg/g) in raw sand smelt fish muscles (Mean values ± standard deviation).

Heavy metal content (µg/g) in the edible portion of raw sand smelt fish is summarized in Fig. 3. The concentrations of lead, cadmium, and arsenic were found to be 1.27 ± 0.03, 0.27 ± 0.02, and 0.70 ± 0.01 µg/g, respectively. Mercury levels were below the detection limit.

### Health risk assessment parameters of heavy metals associated with consuming sand smelt fish muscles

The results of health risk assessment parameters of the analyzed heavy metals (lead, cadmium, mercury, and total arsenic) associated with consuming sand-smelt fish muscles were presented in Table 3. The THQ and HI of analyzed fish muscles were less than 1. The Estimated Daily Intake (EDI) of lead, cadmium, and total arsenic was 0.00101, 0.000214, and 0.000555 mg/kg-d, respectively. Moreover, Target Hazard Quotient (THQ) values of lead, cadmium, and inorganic arsenic were 0.252, 0.214, and 0.185. on the other hand, The Hazard Index (HI) of all examined heavy metals was 0.651. Additionally, the carcinogenic Risk (CR) of examined heavy metals for lead (Pd), cadmium (cd), and total arsenic (As) were 8.55 × 10^−6^, 8.13 × 10^−5^, and 8.33 × 10^−4^, respectively.

**Table 3:** Health risk assessment parameters of heavy metals associated with consuming sand smelt fish muscles.

Heavy metals	EDI	RfD	THQ	CSFo	CR
Lead	1.01 E-03	0.004	0.252	0.0085	8.55 E-06
Cadmium	2.14 E-04	0.001	0.214	0.38	8.13 E-05
Mercury	ND	0.0001	ND	-	ND
Arsenic	5.55 E-04	0.0003	0.185	1.50	8.33 E-04
HI			0.651		

ND: Not detected; Estimated daily intake (EDI, mg/kg-d); RfD: oral reference dose (mg/kg-d); Target Hazard Quotient (THQ) and Hazard Index (HI); CSFo is oral carcinogenic slope factor (mg/kg-d); Carcinogenic risk (CR).

## Discussion

### Nutritional value of sand smelt fish flesh

The obtained results in Fig. 2 showed high moisture content in sand smelt fish, which was about 78.10 ± 0.15% g of fish samples. These results are slightly similar to those obtained by Bilgin et al.^6^ who recorded 78.28 ± 0.20% in sand smelt fish. Likely, Hamaamin and Khidhir^36^ found similar protein content in wild common carp fish (*Cyprinus carpio L.*) was 78.03 ± 03%^36^. As well, a slightly higher percent was obtained by Kalogeropoulos et al.^37^. While, lower finding was observed by El-Sahn et al.^38^, who reported that the moisture content of fresh fish balls processed from *Atherina mochon* was 66.00% in the fresh fish and 66.40% in the beheaded and gutted samples. Furthermore, Albashr et al.^39^ found lesser moisture content in the meat of Harmi (*Barbus luteus*) and Balaout (*Chondrostoma regium*) reaching about 72.13% and 71.63%, respectively. As well, inferior results were detected in the muscles of silver carp (69.19%) and grass carp (74.69%)^40^. On the other hand, Albashr et al.^41^ found lesser protein levels in Bagrus and Muraena fillet (71.36 and 71.10%, respectively). However, El-Lahamy et al.^9^ claimed that the moisture content in sand smelt fish products (fish burger and finger) ranged from 60.22 to 64.66%, respectively. Nevertheless, a lower moisture content of 75.26% may be due to the impact of the marination applied on the fish, which led to a drop in water content due to the ability of the salt to absorb water^42^.

The fat content of sand smelt fish meat was presented in Fig. 2. It was revealed that the fat content in muscles of sand smelt fish is about 1.70 ± 0.03%. These results agreed with those obtained by Bilgin et al.^6^; Cadun et al.^43^ and Eke^44^. Lower results were measured by Hamaamin and Khidhir^36^ who specified that crude fat content was found to be 2.11% in raw sand smelt fish (*Atherina boyeri*) and 3.33% in wild common carp fish. Additionally, the results were lesser than those detected in the muscles of Harmi (*Barbus luteus*) and Balaout (*Chondrostoma regium*) reaching about 5.07% and 4.96%, respectively^39^. Moreover, the obtained results were lower than those investigated in Bagrus and Muraena fillets (3.59 and 4.62%, respectively)^41^. Furthermore, higher fat content was detected in Bizz (*Barbus esocinus*) (2.58–11.73%)^40^. However, El-Lahamy et al.^9^ found that the crude fat content in sand smelt fish products ranged from 2.75 to 5.52% in fish burger and finger, respectively. It may be attributed to the effect of marination in 10% NaCl and 2% acetic acid or 10% NaCl and 3% acetic acid, respectively, which increases the crude fat content to 3.32 and 3.37% as stated by Bilgin et al.^6^.

Moreover, the obtained results in Figure (2) showed that the sand smelt fish are a good source of protein as they contain about 18.53 ± 0.07% fresh samples. These results are closely similar to that determined by El-Lahamy et al.^9^ who observed that the protein content in sand smelt fish products (fish burger) was 18.49% and slightly similar to that obtained by Bilgin et al.^6^ who recorded that the protein content was about 19.64 ± 0.45% of fresh sample and 18.84 ± 0.80% after marination in mixture of 10% NaCl and 2% acetic acid, respectively. Similar results were reached by Hamaamin and Khidhir^36^ who found that protein content in wild and farmed common carp (17.36 and 19.23%, respectively). Likewise, Albashr et al.^41^ observed similar protein content in Harmi (*Barbus luteus*) and Balaout (*Chondrostoma regium*) about 19.74% and 19.98%, respectively. However, a lower protein percent was observed in silver carp *(Hypophthalmichthys molitrix)* (16.82%) and a higher protein content was analyzed in Bizz (*Barbus esocinus*) (21.69%)^40^. On the other hand, Bagrus and Muraena are rich in protein (21.36 and 21.43%, respectively)^41^. Moreover, the results of the current study were more than those assigned by Bilgin et al.^6^_,_who recorded lesser protein contents (16.43 ± 0.33%) after marination (10% NaCl and 3% acetic acid). However, the current study’s protein results closely matched the results obtained by Erkan and Özden^45^_,_ who claimed that the protein content was 17.21% in fresh samples and lower than that studied by Eke^44^ and Çelik et al.^46^.

According to Figure (2), the ash content in fresh samples of sand smelt fish were 1.61 ± 0.12%. These values agreed with that assigned by Bilgin et al.^6^_,_ who recorded ash contents of about 1.67 g/100 g for fresh samples of sand smelt fish. As well, similar ash percent was detected in meat of Balaout (*Chondrostoma regium*) and Harmi (*Barbus luteus*) recording 2.04% and 1.60%, respectively^39^. Moreover, the results were lower than ash percent found in silver carp (1.95%) and higher than those detected in grass carp (0.99%)^36^. Furthermore, lower ash contents were detected either in wild and farmed common carp fish (1.023 and 0.997%, respectively)^36^. Unlikely, Albashr et al.^41^ found higher ash content in Bagrus and Muraena meat (3.01 and 2.55%, respectively). On the other hand, the obtained results were higher than those obtained by El-Lahamy et al.^9^_,_ who determined that the ash content in sand smelt fish products (fish burger and finger) was 0.98 g/100 g fresh sample. In conclusion, the chemical composition of raw sand smelt fish in this study disagreed with that of Ibrahim et al.^47^_,_ who discovered that the proximate composition of raw sand smelt (wet wt.) was 75.49, 13.02, 1.83, and 9.60% for moisture, crude protein, lipid, and ash content, respectively. Although similar findings were reported by Bilgin et al.^6^_,_ who discovered that raw sand smelt flesh contained 1.84% fat, 19.64% protein, 1.67% ash, and 78.28% moisture content. Several factors are responsible for variations in fish’s chemical composition such as the catching environment, age, sex, and season dietary habits^48^.

### Bacteriological examination (log_10_ CFU/g) of Raw sand smelt fish muscles

Microbial growth and its metabolism are the main causes of organoleptic quality deterioration particularly the microbiological changes that occur during the storage of fish and fisheries products. Therefore, Fishery products’ quality and safety are significantly impacted by the spoilage processes by bacteria and fungi from different sources in the food chain^9^.

Results of bacteriological examination of sand smelt meat samples are presented in Table (1). It was revealed that the fresh samples of sand smelt muscles had good microbiological quality because the aerobic plate count (APC) was significantly lower than the maximum limit (7 log_10_ CFU/g) of the microbiological criteria for fresh fish provided by the International Commission of Microbiological Specification for Food (ICMSF)^49,50^. It may be attributed to the freshness of the sand smelt fish and the hygienic conditions used in the handling, and preparation of the samples. The current study findings were incompatible with those assigned by Ibrahim et al.^51^, who recorded higher aerobic plate count (APC), Psychrotrophes, pseudomonas and Aeromonas count in fresh *Tilapia niloticus*, fresh *Mugil Cephalus*, and fresh shrimp, respectively. Additionally, the incidence of pseudomonas spp. in sand smelt fish were lower than other fish species as fresh *Tilapia niloticus*, fresh shrimp and fresh *Mugil cephalous*. Moreover, Higher APC was detected in silver carp *(Hypophthalmichthys molitrix)* (25.80 × 10^5^ CFU/g) and Bizz *(Barbus esocinus)* (8.37 × 10^5^ CFU/g)^40^. However, Shetty and Setty^52^_,_and El-Lahamy et al.^9^ recorded lower values for APC values when they examined the bacteriological status of Indian oil sardines stored in chilled seawater and sand smelt fish burger and finger. They discovered that the total plate count of fresh fish was 3.60 × 10^3^/g at first, but increased to 8.10 × 10^3^ CFU/g when the fish was stored in chilled sea water (2 ± 1 °C). Besides, nearly similar findings are obtained by Abou-Zied et al.^42^_,_ for raw sand smelt fish (*Atherina boyeri*). In terms of coliforms count, results of our study revealed low values within acceptable values assigned by ICMSF, total coliforms limits, 1.0 × 10^2^ CFU/g can be present in the food. Nearly similar results were reported in Algeria by Dib^53^_,_ While higher results were tabulated by Al Shabeeb et al.^54^ for Prawn and Cuttlefish. Interestingly, Pseudomans spp. and Aeromaonas spp. counts in examined sand smelt fish were found lower than count detected in silver carp (59.16 × 10^2^ and 3.325 × 10 CFU/g)^40^.

### Protein deterioration and fat oxidation parameters of Raw sand smelt fish flesh

Data in Table (2) summarized results of different parameters for the shelf life of fresh sand smelt fish which indicated that the pH values of fresh sand smelt fish samples ranged from 6.18 to 6.35 with a mean value of 6.27. These results were in harmony with that determined by El-Sherif and Ibrahim^55^ and Ibrahim and El-Sherif^56^. However, our findings disagreed with that obtained by Bilgin et al.^6^ who recorded a lower pH value of about 6.16. On the other hand, Izci et al.^57^ reported a higher pH value about 6.52 for fresh sand smelt and lower values of about 6.17 for fish chips. This variation may refer to the effect of different treatments and marination of sand smelt fish products and raw flesh.

The Total Volatile Base Nitrogen values (TVB-N) in Table (2) ranged from 15.29 to 16.63 with a mean value of 15.96 ± 0.39 (mg/100 g) these results are similar to that obtained by Bilgin et al.^6^ which determined that TVB-N values were 16.07 mg/100 g. However, higher findings were obtained by Ibrahim et al.^47^ and Abou-Zied et al.^42^, who reported that the TVB-N in fresh sand smelt fish were 14.8 ± 0.24 mg/100 g and 14.27 ± 1.16 mg\100 g, respectively. The elevation in TVB-N value is related to the degradation of protein and other organic compounds found in fish tissues by microbial growth^56^. In the opposite of current study findings, Abou-Zied et al.^42^ recorded lower values for TVB-N, TMA 3.51 ± 0.50 mg\100 g, and TBA 0.63 ± 00.01 mg MDA\kg sample. However, pH values about 6.58 ± 00.17 were higher than our findings. Additionally, our findings differed from those published by Ibrahim et al.^47^ who recorded results of about 6.35, 14.7 mg\100 g, and 0.88 mg MDA/kg for pH, TVB-N, and TBA values, respectively. Similar results were measured by Khidhir^40^ who observrd lower TVB-N values in the meat of Bizz (*Barbus esocinus*) and higher values in silver carp (12.23 and 16.41 mg/100 g, respectively). As well, lower TMA values were detected in Bizz and silver carp meat (2.35 and 5.58 mg/100 g, respectively). On the other hand, TBA values in silver carp meat revealed unacceptable limits (5.20 mg MDA/kg), while similar TBA values were detected in *Barbus esocinus* (0.87 mg MDA/kg) and higher values in common carp (3.48 mg MDA/kg)^40^. The difference in the values of TBA, TVB-N, TMA, FFA, and TMA in different fish species may be attributed to the changes in nutrition, season of harvesting, chemical composition of fat and protein, post harvesting contamination, storage conditions, fatty acid profile, sex, and stress factors.

### Heavy metal content (µg/g) of raw sand smelt fish muscles

Several health benefits acquired by fish eating may be impacted by the presence of toxic metals and metalloids such as lead, cadmium, arsenic and mercury since seafood is a serious accumulator of extensive amounts of heavy metals which potentially threaten human health especially if consumed in toxic quantities. Therefore, the monitoring of metal concentrations in fish meat is important to ensure compliance with food safety regulations and consequent consumer protection^58^. Heavy metal contents (µg/g) of raw sand smelt fish were estimated in Table 3 indicated that the highest concentration among heavy metal was the lead followed by arsenic, and cadmium (1.27 ± 0.03, 0.70 ± 0.01, 0.27 ± 0.02 µg/g), respectively. Our study contents of lead and cadmium exceeded the permissible limits (0.30 mg/kg) described by the Egyptian organization for standardization (EOS)^59^ and European Commission Regulation^27^. However, according to South Africa Department of Health’s regulatory limit for Cd in fish and processed fish is 1.0 mg kg. While, arsenic didn’t exceed acceptable thresholds of the guidelines of about 2.0 mg kg^−1^ assigned by FAO for fish and processed fish meat. Though, the content of mercury was below the detectable limits in all examined samples of raw sand smelt fish^60^.

In the context of lead content, our findings are compatible with those reported by Malak et al.^13^ who claimed that lead is the major heavy metal contaminant in all the examined Egyptian low coast fish species.It may be attributed to the considerable pollution of water from the relatively long-standing human activities in this region. Moreover, the Mediterranean Sea is a semi-enclosed sea, surrounded by thickly polluted and highly industrialized countries, from which large quantities of pollutants enter this sea^61^. On the other hand, higher results of heavy metals particularly for lead and cadmium were indicated by Al-Yousuf et al.^62^ and Jakimska et al.^63^ for Mediterranean sand smelt. Likewise, Helmy et al.^64^ recorded relatively higher levels which exceeded the acceptable limits for both lead, cadmium as well as mercury for different fish and shell fish species in Kalyobia governorate, Egypt. That is mainly owing to anthropogenic activity such as wastewater treatment works, storm water and industrial wastewater.

#### Health risk assessment parameters of heavy metals associated with consuming sand smelt fish muscles

The estimated daily intake (EDI) is used to evaluate the health risk caused by ingestion of sand smelt muscles. Furthermore, the Target Hazard Quotient (THQ) was used to assess the potential non-cancer health risk associated with exposure to a specific heavy metal. In the current study, the THQ values are less than 1, indicating an acceptable risk level. On the other hand, the Hazard Index (HI) represents the combined non-cancer risk from exposure to multiple heavy metals. The HI value of less than 1 suggests an acceptable overall risk. If the Target Hazard Quotient (THQ) and/or Hazard Index (HI) values exceed 1, it suggests a potential risk of non-cancer health effects from exposure to heavy metals. However, if these values are less than 1, the risk is generally considered low. While the THQ and HI values calculated in this study are below 1, this doesn’t definitively guarantee that consuming these fish samples is entirely safe. This is because the assessment only considers exposure from fish consumption and doesn’t account for potential exposure to heavy metals from other dietary sources.

The obtained results for HI and TQH for Pb, Cd, Hg, and total As were similar to those findings reached by Malak et al.^13^ in red porgy, sardine, eel fish, common carp, and grass carp muscles, which indicate that consumption of sand smelt fish muscles does not cause health risk to humans. Similarly, Yang et al.^65^ and Mielcarek et al.^66^ showed that TQH and HI for lead, cadmium, and arsenic in analysed muscles of sardine, carp, and European eels were below 1. In these regards, Soto-Jiménez et al.^67^ revealed that THQs for Cd and Pb in the muscles of sail fish caught from the coast of Mazatlán were < 1. As well, Hasan et al.^68^ found that THQ for lead (Pb), cadmium (Cd), and arsenic (As) were around 1 in *Systomus sarana* (Olive barb), *Mastacembelus armatus* (Tire-track Spiny eel), and *Pethia ticto* (Ticto barb). However, higher TQH (more than 1) for arsenic and less than 1 for mercury, lead, and cadmium in the muscles of flat head grey mullet, Nile tilapia, and African catfish indicate non-carcinogenic health risks for humans.

Regarding to the cumulative effects of all heavy metals (HI), our findings were in consistent with those obtained by Mehmood et al.^71^ who estimated that The total HI for heavy metals (Cd, Cu, Pb, Ni, total Chromium, Cr(VI), and) were to be less than 1 in *Anguilla bengalensis*, *Sperata seenghala*, *Solea solea*, *Labeo rohita*, *Cirrhinus mrigala*. Similiarly, Malak et al.^13^ revealed that HI index of analyzed heavy metals (Pb, Hg, Cd, and Total Ar) in common carp, grass carp, sardine, eel, and red porgy muscles were below 1. This indicate that consumption of sand-smelt fish flesh is safe for human health. Conversely, Hasan et al.^68^ calculated higher HI index (more than 1) in the examined three fish muscles species.

Regarding to Carcinogenic Risk (CR) of analyzed heavy metals in sand-smelt fish muscles (Table 3). The highest value of CR in the examined heavy metals was 10^−6^ which in agreement with permissible limits (10^−4^- 10^−6^) previously stated by United States Environmental Protection Agency (USEPA)^69^ and Sonone et al.^70^ Our findings were in harmony with those calculated by Mielcarek et al.^66^ who observed that CR value of arsenic in European eel fish was 1.82 × 10^−6^. In contrast, Mehmood et al.^71^ found high CR values for lead and chromium and Pb exceeding the permissible limits in the analyzed five fish species. Regardless, Hasan et al.^68^ observed high CR values (ranging from 10^−8^ to 10^−10^) for cadmium, lead, nickel, and arsenic in three fish species caught from Shitalakya River in Bangladesh. Interestingly, consumption of sand-smelt fish muscles. Generally, when CR values become less than 10^−6^ are considered as minimal carcinogenic risk but When their values are higher than 10^−4^ are regarded as unacceptable cancer risk^35,72,73^. Consequently, all analyzed heavy metals may cause carcinogenic effect for consumers by overconsumption of sand-smelt fish muscles. So, our study suggests that the environmental health management authorities should make continuous surveillance for heavy metals in fish especially those catches from contaminated rivers and seas to decrease the possible carcinogenic effect of consuming such species of fish.

## Conclusion

Sand smelt fish is affordable, nutritive, and has good shelf life indicators, concerning heavy metal content, Target Hazard Quotient (THQ) and Hazard Index (HI) values were below 1, suggesting a low risk of non-carcinogenic effects. Carcinogenic risks were also considered low. However, this doesn’t guarantee that consuming these fish samples is entirely safe. Consequently, sand smelt fish meat can be widely incorporated safely in Egyptian markets. However, continuous monitoring of heavy metal levels is recommended to ensure long-term food safety and great attention should be paid to proper application of sufficient cooking programs and others strategies adopted for reduction of these toxic heavy metal levels or limitation of daily uptake thresholds to ensure public health of consumers to maximize the benefits from consumption of such affordable and functional food items.

## Data Availability

Data will be made available on request from corresponding authors.
